# Meta-Analysis of the Relationship between the *APOE* Gene and the Onset of Parkinson's Disease Dementia

**DOI:** 10.1155/2018/9497147

**Published:** 2018-10-14

**Authors:** Suisui Pang, Jia Li, Yingyu Zhang, Jiajun Chen

**Affiliations:** Department of Neurology, China–Japan Union Hospital of Jilin University, No. 126, Xian Tai Road, Changchun, Jilin 130033, China

## Abstract

**Purpose:**

To clarify the relationship between certain genotypes or alleles of the *APOE* gene and the onset risk of Parkinson's disease dementia (PDD).

**Methods:**

The PubMed, Cochrane, Embase, CBM, CNKI, and Wanfang databases were searched to identify all case-control studies and cohort studies published before October 30, 2017, that investigated the association between the *APOE* gene and the onset of PDD. Manual information retrieval was also performed. All studies that met the quality requirements were included in a meta-analysis performed using RevMan 5.3 software.

**Results:**

The meta-analysis included 17 studies, with a total of 820 patients in the PDD group and 1,922 in the non-PDD group. The influence of the *APOE* gene on PDD onset was analyzed from three aspects: five genotypes vs. *ε*3/3, *ε*2+/*ε*4+ vs. *ε*3/3, and *ε*4+ vs. *ε*4−. The risk factors for PDD may include the genotypes *ε*3/4 (OR 1.47, 95% CI 1.14–1.89) and *ε*4/4 (OR 2.93, 95% CI 1.20–7.14). In patients with PDD, there was no significant difference in the distribution of *ε*2+ vs. *ε*3/3 (OR 1.35, 95% CI 0.97–1.87, *P*=0.07). The risk of PDD was 1.61 times greater in *ε*4+ compared with *ε*3/3 (OR 1.61, 95% CI 1.24–2.08, *P*=0.0003). As the results indicated that *ε*2+ did not play a role as a risk factor or a protective factor, we divided the population into *ε*4+ and *ε*4− for the meta-analysis and found that, among patients with Parkinson's disease, the dementia risk of those with *ε*4+ was 1.72 times greater than that of those with *ε*4− (OR 1.72, 95% CI 1.41–2.10, *P* < 0.00001). Subgroup analysis in accordance with different geographical regions revealed that *ε*4+ was a risk factor for PDD in people from all regions.

**Conclusions:**

Among the *APOE* genotypes, *ε*2+ is neither a risk factor nor a protective factor for PDD, while *ε*4+ is a risk factor for PDD. The present results are applicable to Asian, European, and American patients with Parkinson's disease. Regarding the single *APOE* genotypes, *ε*3/4 and *ε*4/4 may be risk factors for PDD; however, further studies with large sample sizes are needed to verify this.

## 1. Introduction

Parkinson's disease (PD) is a common neurodegenerative disease among middle-aged and older adults. The major clinical features of PD include motor symptoms (such as static tremor, bradykinesia, myotonia, and postural balance disturbance), as well as nonmotor symptoms (such as disturbances of olfactory sensation and other senses, sleep disorders, autonomic dysfunction, and cognitive disorders). Parkinson's cognitive disorders are a common nonmotor symptom of PD, and these can be divided into mild cognitive impairment and Parkinson's disease dementia (PDD). An epidemiological investigation performed in 2005 showed that dementia develops in 24–31% of patients with PD and that PDD accounts for 3-4% of patients with all types of dementia [1]. PDD can have a strong impact on the quality of life and social function of patients and can increase the mortality and disability rates [2]; this increases the burden of carers, prolongs the duration of hospitalization, increases hospitalization costs, and causes substantial burdens to family and society.

The clinical features of PDD include insidious onset and slowly developed deficits of attention, executive function, visual spatial function, and memory, accompanied by illusion, delusion, indifference, and other spiritual and emotional changes [3]. The pathogenesis is still unclear; however, PDD may be caused by various pathologic changes, such as an increase in the number of Lewy bodies in brain tissue, neurofibrillary tangles, senile plaque formation, microvascular lesions, and the presence of argyrophilic inclusion bodies [4–8]. The risk factors for PDD are also diverse and may include demographic characteristics and living habits such as advanced age, lower educational level, and smoking; the risk of PDD may also be increased in those with akinetic-rigid motor symptoms, those with nonmotor symptoms like mild cognitive impairment, rapid eye movement sleep behavior disorder, and illusion, and those with changes in biologic tumor markers such as low serum epidermal growth factor and low uric acid [9]. With technological developments, researchers have begun to explore the risk factors for PDD at a genetic level, and the *APOE*, *MAPT*, *SNCA*, *GBA*, *LRRK2*, and *COMT* genes have been found to play a role in the onset and development of PDD [10].

The risk factors for the onset of PDD are likely to be related with the presence of specific genes, and the presence of a certain genotype or an allele may predict whether the risk of PDD is increased in patients with PD. This would enable the risk factors for the onset of PDD to be predicted through testing for related genes, and people identified as being at high risk of PDD could promptly commence tracking and prevention therapies to prevent PDD and suspend its progress. Regarding the research into various genes related to the risk factors for the onset of PDD, a greater number of studies have evaluated the *APOE* gene than any other gene, and the *APOE* gene is generally regarded as the gene that has the largest influence on dementia and a stronger predictability compared with other genes.

The *APOE* gene has *ε*2, *ε*3, and *ε*4 alleles, which can be classified into six different genotypes: *ε*2/*ε*2, *ε*2/*ε*3, *ε*2/*ε*4, *ε*3/*ε*3, *ε*3/*ε*4, and *ε*4/*ε*4. These genotypes can be divided into the E2 phenotype (*ε*2/2 and *ε*3/2), E3 phenotype (*ε*3/3), and E4 phenotype (*ε*4/3, *ε*4/2, and *ε*4/4), among which the E3 phenotype is the most common and is referred to as the wild type [11]. These three phenotypes correspond to their respective protein isoforms (E2, E3, and E4) [12], and these three protein isoforms are collectively called apolipoprotein E (*APOE*) [13]. In the central nervous system, *APOE* can influence cholesterol/lipid homeostasis, synaptic function, glycometabolism, neurogenesis, mitochondrial function, tau protein phosphorylation, neuron atrophy, neuroinflammation, and the metabolic and gathering pathways of *β*-amyloid protein (A*β*) [14–16]. *APOE* can also protect the central nervous system by reducing its oxidative stress and inflammatory response level, resulting in cerebral protection [17]. Furthermore, *APOE* can stimulate neural stem cells to enhance their survival through the conduction path of the extracellular-signal-regulated kinase signal [18]. Different genes may lead to different *APOE* functions, affecting the abovementioned biochemical reaction processes and causing cognition impairment.

At present, it is widely believed that the *APOEε*2 allele protects the central nervous system, and a longitudinal study found that *APOEε*2 effectively reduces damage to the parts of the brain that control daily function and episodic memory [19]. In contrast, *APOEε*4 causes damage to the central nervous system that can increase the risk of cognitive disorder and is one of the major risk factors for dementia [20]. Many studies have investigated the effect of the *APOE* gene on the risk of onset of PDD. A case-control study investigating the relationships between PDD and different *APOE* genotypes found no obvious differences in *APOE* genotypes and gene frequency between patients with versus without PDD [21]. In contrast, one study reported that *APOEε*2+ and *ε*4+ might carry a higher risk of PDD [22], while another study verified that *APOEε*4 increases the risk of PDD and that *ε*2 has no relationship with dementia development in patients with PD [23]. The conclusions of other studies vary due to differences in race, age, and sex; furthermore, the study results are also influenced by research techniques, diagnostic criteria, and sample size. Hence, there is a need for an objective quantitative synthesis of the currently available research results to further define whether the *APOE* gene is related to the risk factors for the onset of PDD and to define its risk level.

The present meta-analysis was performed to make a quantitative synthesis and comprehensive assessment of published materials on the association between the *APOE* gene and the onset of PDD. The aim of the present meta-analysis was to provide a more objective evidence-based medicine foundation for the relationship between different *APOE* genotypes and the risk factors for the onset of PDD.

## 2. Materials and Methods

### 2.1. Data Retrieval

A method combining subject and free terms was applied to comprehensively and systematically search the PubMed, Cochrane, Embase, CBM, CNKI, and Wanfang databases for case-control studies and cohort studies published before October 30, 2017, that investigated the relationship between the *APOE* gene and the onset of PDD. The references of retrieved articles, conference literature, and gray literature were also searched manually. The search words were Parkinson disease, Parkinson's disease, primary parkinsonism, parkinsonism, primary, paralysis agitans, Parkinson's disease, Parkinson dementia complex, apolipoprotein *E*, apoprotein *E*, *APOE*, APO-E, APO *E*, AD2, LPG, LDLCQ5, dementia, cognition disorders, cognitive defect, dementias, demention, amentia, amentias, case-control study, and cohort studies.

### 2.2. Inclusion and Exclusion Criteria

Inclusion criteria were as follows: (1) observational study investigating the relationship between the *APOE* gene and the onset of PDD; (2) explicit clinical diagnosis of PD (made using the diagnostic criteria of PD from UK Brain Bank, Calne criteria, or diagnostic criteria of the First National Symposium on Extrapyramidal Diseases in China) or pathological diagnosis; (3) *APOE* genotype recorded; (4) at least one method used to assess dementia; (5) complete description of the results, and the odds ratio (OR) and 95% confidence interval (CI) of the case and control groups could be obtained either directly or indirectly; (6) case-control study or cohort study; (7) published in Chinese or English; (8) full text could be obtained, or the authors could provide the requisite information and data; and (9) published or unpublished materials before October 30, 2017. Exclusion criteria were as follows: (1) failure to match the research aim (study did not include patients with PD, or the investigated gene was not *APOE*); (2) diagnostic criteria of PD were not stated clearly, or unspecialized diagnostic criteria were used; (3) incomplete gene detection records; (4) the method used to assess dementia was not described; (5) abstract, literature review, case report, seminar, or repetitively published literature; for repetitively published literature, the most recent article or the article with most complete data was selected; and (6) full text could not be obtained, or sample data were not complete or clear and requisite information and data could not be acquired after contacting the author.

### 2.3. Literature Quality Assessment

The Newcastle-Ottawa Scale was used to assess the methodological quality of the included studies [24]. For case-control studies, this comprised the determination of (1) adequate case definition, (2) representativeness of cases, (3) selection of control, (4) definition of control, (5) comparability of case and control groups, (6) exposure, (7) whether there were identical exposure methods for cases and controls, and (8) nonresponse rate. For cohort studies, this comprised the determination of (1) representativeness of the exposed cohort, (2) selection of the unexposed cohort, (3) determination of exposure, (4) whether the study subjects had an ending event that occurred before the study began, (5) comparability of the cohorts, (6) evaluation of the ending event, (7) whether follow-up was sufficient, and (8) integrality of follow-up examinations.

Exposure was defined as the allele or genotype of the *APOE* gene, and the exposure assessment method was defined as the method used to detect the gene. Each item that met one of the abovementioned criteria was represented by ∗, and each ∗ was equivalent to 1 point, giving a potential total of 9 points. Higher scores indicated higher quality studies; studies with a score of 6 points or higher were included in the present meta-analysis.

### 2.4. Data Extraction

The following data were extracted and tabulated: first author, publication date, country of the study population, race, age, diagnostic criteria of PD, diagnostic criteria of dementia, study design, sample capacity, and genotype distributions of the case and control groups. The literature screening, quality assessment, and data extraction were completed by two researchers, and disagreements were resolved via discussion with a third researcher.

### 2.5. Statistical Analysis

RevMan 5.3 software was used to analyze the relationship between the *APOE* gene and the onset of PDD and to calculate the OR and 95% CI for the analyses of the five genotypes (*ε*2/*ε*2, *ε*2/*ε*3, *ε*2/*ε*4, *ε*3/*ε*4, and *ε*3/*ε*4) vs. *ε*3/3, *ε*2+/*ε*4+ vs. *ε*3/3, and *ε*4+ vs*. ε*4−. The *Q* value and *I*^2^ were used to test the heterogeneity. *P* < 0.10 was considered to indicate heterogeneity between combined studies. *I*^2^ values of 0–25% indicated no heterogeneity, 25–50% indicated mild heterogeneity, 50–75% indicated moderate heterogeneity, and 75–100% indicated major heterogeneity [25, 26]. The statistical analysis method was selected in accordance with the heterogeneity results; when there was no heterogeneity, the Mantel–Haenszel fixed-effect model [27] was used for data consolidation analysis, while the DerSimonian–Laird random-effect model [28] was used in other cases. *Z* was used to test and calculate the significance of the OR value and was the criterion used to evaluate risk correlation. The applied inspection level was *a* = 0.05, and *P* < 0.05 was considered to indicate a significant difference. Heterogeneity tests were initially done within each group; when heterogeneity was detected, the source of the heterogeneity was investigated via subgroup analysis. The single removal method was applied in the sensitivity analysis to test the stability of the results. Funnel plots were used to test for publication bias.

## 3. Results

### 3.1. Data Retrieval

**Figure 1:**
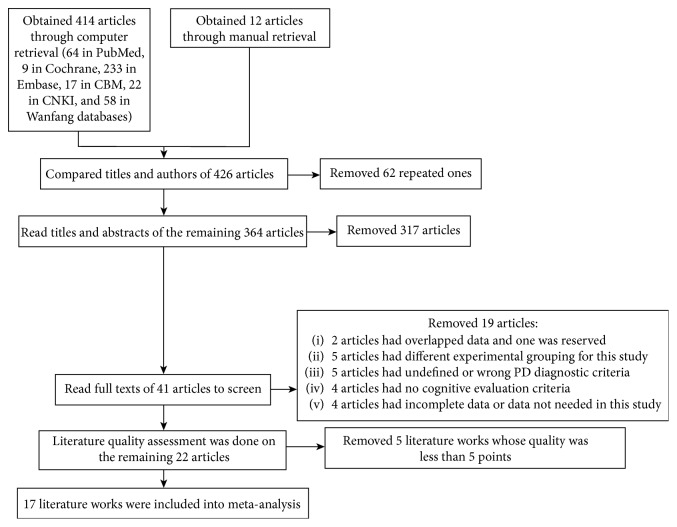
Flow chart of the study retrieval process.

There were 426 articles retrieved; screening of the titles and abstracts resulted in the exclusion of 62 articles that were repeated literature, six that were conference literature, and 316 that were incompatible with the research contents of the present analysis. Of the remaining 42 articles, the original text of one article could not be obtained, two articles reported the same experiment (the one with more complete data was included), the experimental grouping in five articles differed from that used in the present meta-analysis, five articles had used undefined or incorrect diagnostic criteria for PD, four articles had no cognitive evaluation criteria, four articles had incomplete or irrelevant data, and the quality assessment score of five articles was less than 6 points. A final total of 17 articles were included in the meta-analysis (the retrieval process is shown in Figure 1).

### 3.2. Essential Features of the Included Studies

All 17 included studies investigated the relationship between dementia in patients with PD who carried the *ε*4 genotype (*ε*4+) and those without the *ε*4 genotype (*ε*4−) [4, 21–23, 30–42]. Only 10 included studies comprehensively evaluated the relationships between all six genotypes of the *APOE* gene and PDD [21–23, 30–36]. Relevant data are shown in Table 1.

### 3.3. Meta-Analysis Results

#### 3.3.1. Risk Factors for the Onset of PDD for Each Genotype

**Figure 2:**
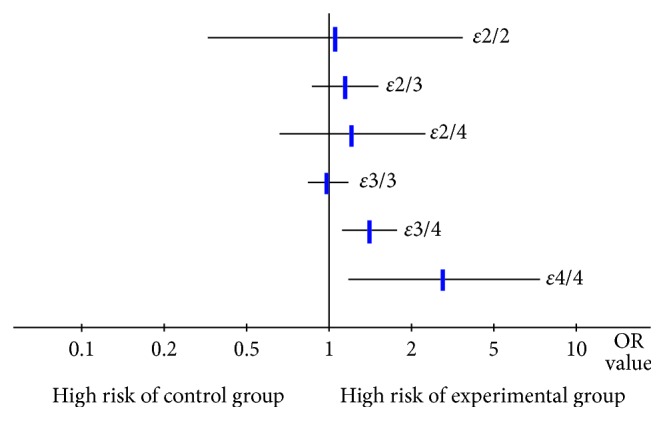
Risk of Parkinson's disease dementia in those with the five *APOE* genotypes compared with the *ε*3/3 genotype (assessed in 10 studies).

In the 10 included studies that investigated all genotypes of the *APOE* gene, the frequencies of some genotypes were low and the event counts were 0, and thus, it was impossible to calculate the OR values separately. Therefore, we calculated OR values and 95% CIs for the five genotypes (*ε*2/*ε*2, *ε*2/*ε*3, *ε*2/*ε*4, *ε*3/*ε*4, and *ε*4/*ε*4) vs. *ε*3/3. As shown in Figure 2, compared with patients with PD who had the *ε*3/3 genotype, there was a significantly greater risk of dementia in those with genotypes *ε*3/4 (OR 1.47, 95% CI 1.14–1.89) and *ε*4/4 (OR 2.93 95% CI 1.20–7.14), while there was no difference in the risk of PDD between those with the *ε*3/3 genotype and those with the genotypes *ε*2/2 (OR 1.07, 95% CI 0.33–3.48), *ε*2/3 (OR 1.17, 95% CI 0.85–1.61), and *ε*2/4 (OR 1.25, 95% CI 0.63–2.47).

#### 3.3.2. Risk Factors for the Onset of PDD for *ε*2+ and *ε*4+

**Figure 3:**
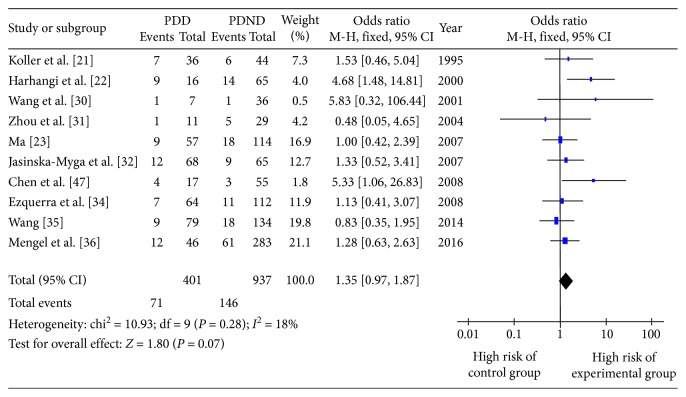
Forest plot for the risk of Parkinson's disease dementia onset in *ε*2+ patients assessed in 10 studies.

**Figure 4:**
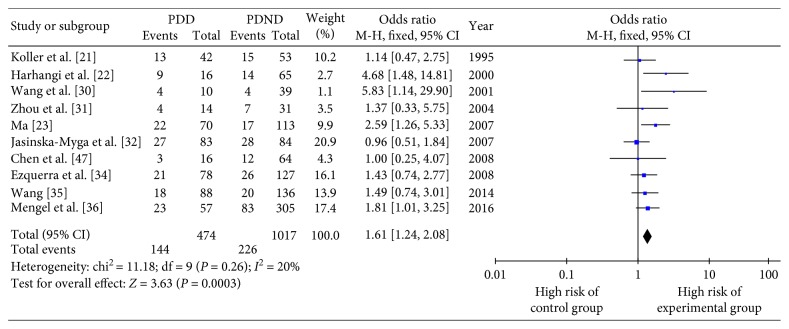
Forest plot for the risk of Parkinson's disease dementia onset in *ε*4+ patients assessed in 10 studies.

The OR and 95% CI for *ε*2+/*ε*4+ vs. *ε*3/3 in 10 included studies are shown in Figures 3 and 4, respectively. There was no obvious heterogeneity (*Q* testing, *P* > 0.10), and so the fixed-effect model was selected. The combined OR value in Figure 4 shows that the risk of PDD development in *ε*4+ patients was 1.61 times greater than that in those with the *ε*3/3 genotype (OR 1.61, 95% CI 1.24–2.08, *P*=0.0003).  Figure 3 shows that those with the *ε*3/3 genotype had a similar risk of PDD development compared with *ε*2+ patients (OR 1.35, 95% CI 0.97–1.87, *P*=0.07).

#### 3.3.3. PDD Onset Risk of *ε*4+ versus *ε*4− Patients

**Figure 5:**
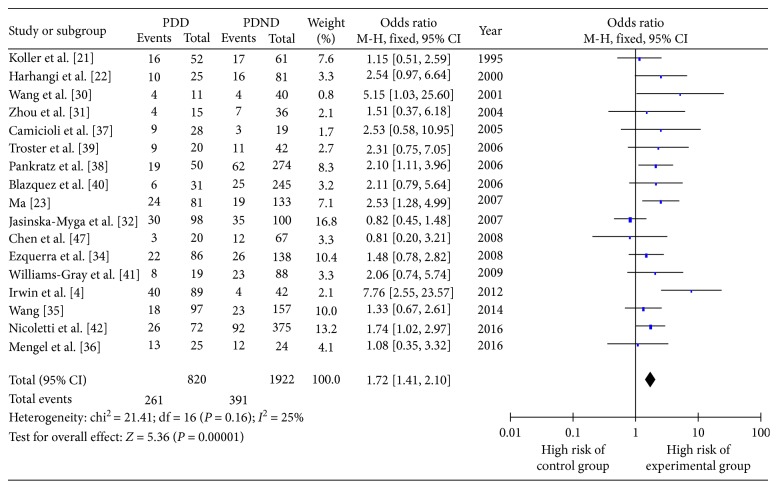
Forest plot for the risk of Parkinson's disease dementia onset in *ε*4+ versus *ε*4− patients assessed in 17 studies.

This part of the meta-analysis included 17 studies, and the OR and 95% CI for *ε*4+ vs. *ε*4− are shown in Figure 5. As heterogeneity = 0.16 and *I*^2^ = 25%, the fixed-effect model was used. The combined OR value in Figure 5 shows that the risk of PDD onset in *ε*4+ patients was 1.72 times greater than that in *ε*4− patients (OR 1.72, 95% CI 1.41–2.10, *P* < 0.00001), indicating that carrying the *ε*4 genotype was a significant risk factor for the development of PDD.

### 3.4. Subgroup Analysis

To determine whether there were regional differences in the influence of the *APOE* gene on the risk of PDD onset, we performed a subgroup analysis in accordance with the regional distributions of patients.

#### 3.4.1. Influence of Regional Distribution on PDD Onset Risk of *ε*2+/*ε*4+

**Figure 6:**
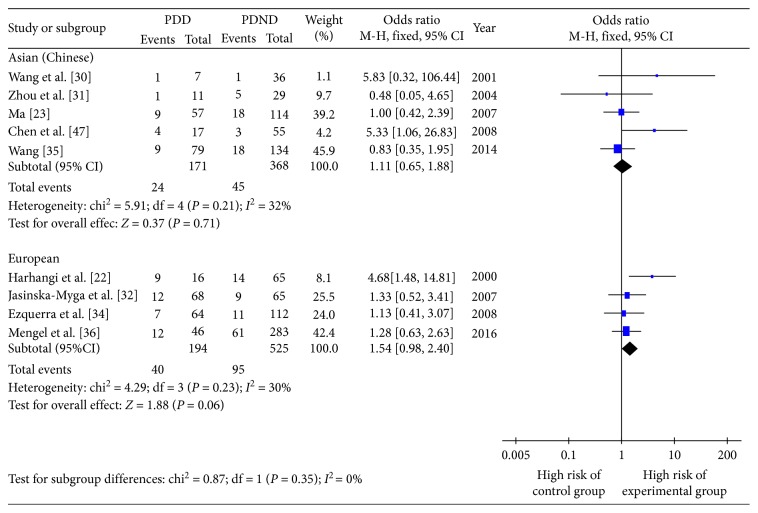
Forest plot for the influence of regional distribution on Parkinson's disease dementia onset risk of *ε*2+ patients.

**Figure 7:**
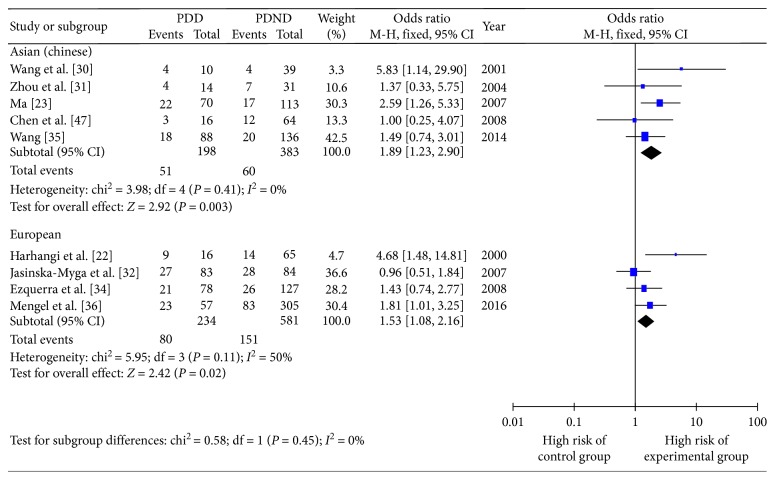
Forest plot for the influence of regional distribution on Parkinson's disease dementia onset risk of *ε*4+ patients.

In accordance with the geographic distribution of the study populations, the 10 studies were divided into five studying Asian patients (Chinese), four studying European patients, and one studying American patient. In both Asian and European patients, *ε*2+ was not a risk factor for PDD development compared with patients carrying the *ε*3/3 genotype (Figure 6). However, for Asian patients, the risk of PDD was 1.89 times greater in *ε*4+ compared with *ε*3/3 (OR 1.89, 95% CI 1.23–2.90, *P*=0.003); for Europeans, the risk of PDD was 1.53 times greater in *ε*4+ compared with *ε*3/3 (OR 1.53, 95% CI 1.08–2.16, *P*=0.002). All subgroups had no heterogeneity (Figure 7).

#### 3.4.2. Differences in PDD Onset Risk between *ε*4+ and *ε*4− Patients in Different Regions

**Figure 8:**
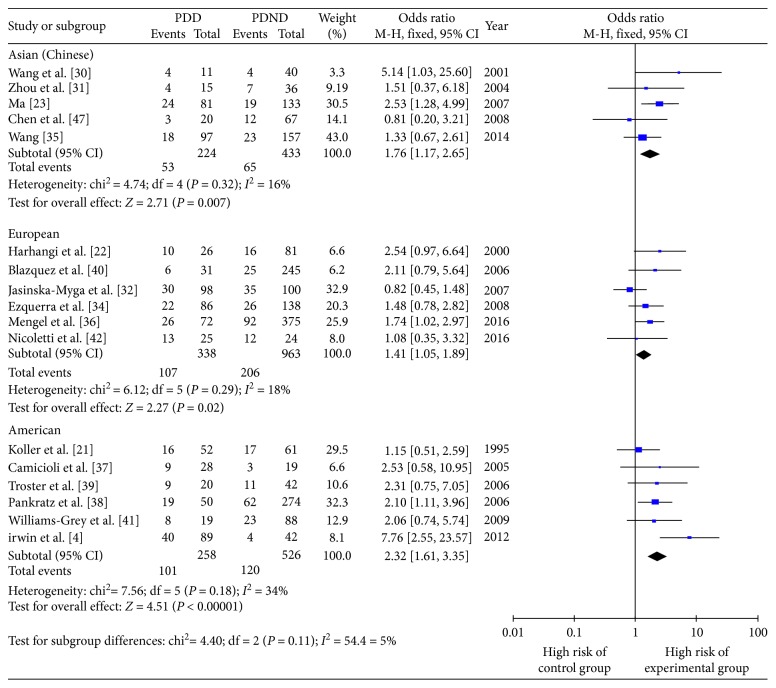
Forest plot for the influence of geographical distribution on Parkinson's disease dementia onset risk of *ε*4+ patients compared with *ε*4− patients.

In accordance with the geographic distribution of the study populations, the 17 studies were divided into five studying Asian patients (Chinese), six studying European patients, and six studying American patients.  Figure 8 shows that, in Asia, Europe, and America, carrying the *ε*4 genotype was a risk factor for PDD development, but the degree of risk varied in different regions; the risk of PDD onset in *ε*4+ patients compared with *ε*4− patients was increased by a factor of 1.46 in Asian patients (OR 1.76, 95% CI 1.17–2.65, *P*=0.007), a factor of 1.41 in European patients (OR 1.41, 95% CI 1.05–1.89, *P*=0.02), and a factor of 2.32 in American patients (OR 2.32, 95% CI 1.61–3.35, *P* < 0.00001).

### 3.5. Sensitivity Analysis

In Section (3.4), we performed analyses of *ε*2+ vs. *ε*3/3, *ε*4+ vs. *ε*3/3, and *ε*4+ vs. *ε*4−. After removing the studies included in the analyses of *ε*4+ vs. *ε*3/3 and *ε*4+ vs. *ε*4−, we performed a meta-analysis of the remaining studies; there were no obvious changes in the combined OR values, and all had statistical significance. Moreover, we did not identify any individual studies that had brought significant heterogeneity into the analysis of various studies. Removing the study published by Wang in 2014 [35] from the analysis of *ε*2+ vs. *ε*3/3 changed the result from having no statistical significance (OR 1.35, 95% CI 0.97–1.87, *P*=0.07) to having statistical significance (OR 1.48, 95% CI 1.04–2.11, *P*=0.03); however, the quality of this study was high, the diagnosis and gene detection methods were standard, the experiment design was reasonable, and the results were reliable [35], and so we concluded that this study should not be removed blindly. We considered that the reason that this study made such an impact on the stability of the analysis was that it had a large sample size and thus its proportion of the overall result was large, which led to a change in the overall result after its removal. To evaluate the influence of sample size, we performed separate meta-analyses on the large sample size group (experimental and control groups both >50) and small sample size group (experimental or control groups <50) and found that the result of the large sample size group was stable and had no statistical significance or heterogeneity (OR 1.04, *P*=0.87, *P* for heterogeneity = 0.90, *I*^2^ = 0%).

### 3.6. Publication Bias Analysis

**Figure 9:**
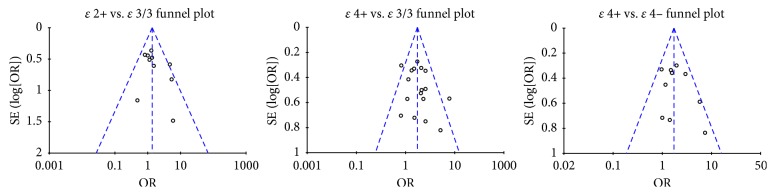
Funnel plots of *ε*2+ vs. *ε*3/3, *ε*4+ vs. *ε*3/3, and *ε*4+ vs. *ε*4−.

Visual inspection revealed that the funnel plots of *ε*2+ vs. *ε*3/3 and *ε*4+ vs. *ε*3/3 were basically symmetrical, with all points evenly dispersed on both sides of the central line and basically located within the 95% CI and no unfilled corners. Hence, we considered that the possibility of bias was not large. Inspection of the funnel plot of *ε*4+ vs. *ε*4− revealed that the symmetry was good, but that two studies were located outside the 95% CI, indicating that there might be a degree of bias; however, separate removal of these two studies showed that their removal exerted no influence on the result (Figure 9).

## 4. Discussion

The present meta-analysis included 17 studies, comprising 820 patients in the experimental group (PDD group) and 1,922 in the control group (non-PDD group). The PDD onset risks of patients with different genotypes of the *APOE* gene were analyzed from three aspects: five genotypes vs. *ε*3/3, *ε*2+/*ε*4+ vs. *ε*3/3, and *ε*4+ vs. *ε*4−. It was revealed that the *ε*3/4 and *ε*4/4 genotypes may be risk factors for PDD. *ε*2+ was neither a risk factor nor a protective factor for the development of PDD compared with the *ε*3/3 genotype, and the distribution of *ε*2+ was similar in the PDD and non-PDD groups. The incidence of *ε*4+ was significantly greater in the PDD group than the non-PDD group, suggesting that *ε*4+ was a risk factor for PDD onset. As *ε*2+ had no role as a risk factor or a protective factor in the development of PDD, we divided the patients into *ε*4+ and *ε*4− for the meta-analysis, which revealed that the risk of PDD onset was 1.72 times greater in patients who are *ε*4+ compared with *ε*4− patients, but the risks varied slightly in accordance with the geographical region; the increased risk of PDD onset in those who were *ε*4+ compared with those who were *ε*4− was the highest in American patients (2.32 times greater), while it was 1.76 times greater in Asian patients and 1.41 times greater in European patients.

The mechanism by which different *APOE* genotypes influence dementia development in patients with PD is still unclarified, although many studies have investigated the mechanism by which *APOEε*4 leads to dementia. *APOEε*4 participates in the mechanism of dementia via the following four aspects: first, A*β* retention can form age pigmentation and vascular amyloidosis and thus lead to dementia. *APOE* adjusts the combination of A*β* through lipidation [43], and it combines with A*β* in the form of a molecular chaperone to influence the elimination of A*β*. The ability of *ε*4+ to eliminate A*β* is weaker than that of *ε*3/3 [44]. Second, tau albumen participates in normal apoptosis and maintains the stability of the cell, and the impairment of the microtubule assembly ability of unusually phosphorylated tau albumen can lead to the destruction of nerve cells. The albumens in the E3 and E2 phenotypes combine with tau albumen through the Cys residue to form stable compounds, protecting the structure of tau albumen and preventing it from undergoing abnormal phosphorylation; however, the residue in the E4 phenotype is minimal, and its ability to combine with tau albumen is weak, leading to abnormal phosphorylation [45]. Third, different phenotypes of the *APOE* gene can participate in the immune adjustment of the central nervous system; the immune response of the central nervous system in *ε*4+ is stronger than that in *ε*3+, and excessively strong immune responses can lead to brain injury and dementia [46]. Fourth, the *APOE* gene subtype can play a regulatory effect in the injury and repair of synapses, and a decline in the number of dendrites in the hippocampus of *ε*4+ may be related to dementia [47].

The present meta-analysis revealed that *APOEε*4 is one of the risk factors for PDD, and this information can be used to guide the therapeutic direction in patients with PD. Detection of the *APOE* gene can predict the risk of PDD onset; high-risk patients can then be more closely monitored, intervening measures (such as controlling the risk factors) can be implemented to prevent PDD, and PDD can be diagnosed and treated in the early stage so that the disease progression can be postponed, patients' quality of life can be improved, social and family burdens can be relieved, and the mortality rate can be lowered. It is already known that the onset of Alzheimer's disease (AD) is related to the *APOE* gene, and the present results indicate that this gene is also a risk factor for the onset of PDD, suggesting that the pathogeneses of PDD and AD may be similar. Although there are only few studies on PDD, we may be able to use the relatively better understood pathogenesis and treatment of AD as a reference to provide ideas for research on PDD. In addition, as the pathogenic factors of AD and PDD may be similar, it is possible that the treatment methods used for AD are applicable to PDD. These theories should be investigated in subsequent studies, for which the results of the present meta-analysis can provide theoretical foundations.

Compared with two previous meta-analyses [41, 48], the present meta-analysis used stricter criteria concerning the inclusion, exclusion, and quality of studies. We also included new studies that had not been published when the previous meta-analyses were performed and excluded studies with poor experimental designs, Newcastle-Ottawa Scale scores of less than 6 points, and no definite diagnostic descriptions of PD and PDD. The present meta-analysis also had some limitations. First, although the diagnostic criteria of PD and PDD have been refined, the diagnostic criteria have not been unified; for example, various studies used only the PD diagnostic criteria of the UK Brain Bank or used PDD criteria to directly assess cognitive disorders. Second, the occurrence and severity of cognitive disorder can be influenced by age, education level, smoking history, living habits, and the presence of other genes that may cause dementia; however, no original data have been studied comprehensively, and thus, subgroup analysis or metaregression analysis cannot be performed. Third, the sample size of some included studies was small, and the event counts of many genotypes with small occurrence frequencies were 0, so the OR values of each genotype could not be calculated; therefore, sensitivity analysis and heterogeneity testing could not be conducted, and the results could not be systematically assessed. Hence, the risk of PDD in patients with certain genotypes should be predicted from the combined sample. Fourth, there were fewer community-based control studies and more hospital-based studies; thus, the samples may not be representative of the general population of patients with PD.

The present meta-analysis investigated the risk of PDD onset in relation to the presence of the *APOE* gene and revealed some limitations that may provide future research directions. A reasonable PDD diagnostic method is urgently required, as no studies have investigated the effectiveness of the currently available diagnostic criteria for PDD, so many studies have used unsuitable criteria such as the diagnostic and statistical manual of mental disorders (DSM) and the mini-mental state examination. In addition, future studies should enhance the representativeness and credibility of sample populations, adopt multicenter, multiracial, and larger sample sized community-based control or cohort studies, and further evaluate the link between the *APOE* gene and the risk of PDD onset among people of different ages and education levels.

## 5. Conclusions

Among the *APOE* genotypes, *ε*2+ is neither a risk factor nor a protective factor for PDD onset, while *ε*4+ is a risk factor for PDD. The present findings are especially applicable to Asian, European, and American patients with PD. Regarding single *APOE* genotypes, *ε*3/4 and *ε*4/4 may be risk factors of PDD, but studies with large sample sizes are needed to verify this.

## Figures and Tables

**Table 1:** Data extraction table.

Researcher	Time	Country	Experimental group (PDD group)						Control group (PDND group)						Experimental group		Control group		PDD diagnostic criteria	Dementia evaluation method	Experimental method	Sample capacity (experimental group and control group ≥ 50)	Patient source	Average age	Literature quality
			2/2	2/3	2/4	3/3	3/4	4/4	2/2	2/3	2/4	3/3	3/4	4/4	*ε*4+	*ε*4−	*ε*4+	*ε*4−							
Koller et al. [21]	1995	America	1	6	3	29	12	1	0	6	2	38	14	1	16	36	17	44	Calne criteria	DRS	Case-control study	Yes	Research center	PD67.4, PDD74.7	8
Harhangi et al. [22]	2000	Netherlands	1	8	1	7	9	0	0	14	2	51	13	1	10	16	16	65	Calne criteria	DSM	Cohort study	No	Community	PD75.8, PDD82.1	7
Wang et al. [30]	2001	China	0	1	0	6	4	0	0	1	0	35	4	0	4	7	4	36	Diagnostic criteria of National Symposium on Extrapyramidal Diseases in 1984	DSM	Case-control study	No	Hospital	PD66.13, PDD71.09	6
Zhou et al. [31]	2004	China	0	1	0	10	3	1	2	3	0	24	6	1	4	11	7	29	Diagnostic criteria of National Symposium on Extrapyramidal Diseases in 1984	DSM	Case-control study	No	Hospital	平均Averagely 67.4	6
Camicioli et al. [37]	2005	Canada													9	19	3	16	Pathology	DSM	Cohort study	No	Hospital	PD77.5, PDD78.1	7
Pankratz et al. [38]	2006	America													19	31	62	212	UK Brain Bank	MMSE	Case-control study	No	Community	Averagely 60.9	7
Troster et al. [39]	2006	America													9	11	11	31	Calne criteria	DRS	Case-control study	Yes	Research center	Averagely 68.6	7
Blazquez et al. [40]	2006	Spain													6	25	25	220	UK Brain Bank	MMSE	Case-control study	No	Hospital	Averagely 71.1	7
Ma [23]	2007	China	0	9	2	48	19	3	1	17	2	96	16	1	24	57	19	114	UK Brain Bank	DSM	Case-control study	No	Hospital	PD68.38, PDD69.72	8
Jasinska-Myga et al. [32]	2007	Poland	1	11	3	56	24	3	1	8	7	56	25	3	30	68	35	65	UK Brain Bank	DSM, MMSE	Case-control study	Yes	Hospital	PD61.7, PDD71.4	8
Tong [33]	2008	China	0	4	0	13	3	0	0	3	0	52	12	0	3	17	12	55	UK Brain Bank	DSM, MMSE	Case-control study	Yes	Hospital	PD70.35, PDD75.44	7
Ezquerra et al. [34]	2008	Spain	0	7	1	57	20	1	0	11	0	101	26	0	22	64	26	112	UK Brain Bank	PDD diagnostic criteria	Case-control study	No	Hospital	PD56, PDD58.3	7
Williams-Gray et al. [41]	2009	America													8	11	23	65	UK Brain Bank	DSM, MMSE	Cohort study	Yes	Community	Unknown	7
Irwin et al. [4]	2012	America													40	49	4	38	UK Brain Bank	DSM	Case-control study	No	Research center	PD80, PDD79	7
Wang [35]	2014	China	1	8	0	70	17	1	0	18	3	116	20	0	18	79	23	134	UK Brain Bank	PDD diagnostic criteria	Case-control study	No	Hospital	PD65.20, PDD67.95	8
Mengel et al. [36]	2016	Germany	0	12	3	34	22	1	5	56	9	222	81	2	26	46	92	283	UK Brain Bank	MDS-TFC	Case-control study	Yes	Research center	Averagely 66.7	6
Nicoletti et al. [42]	2016	Italy													13	12	12	12	UK Brain Bank	MMSE	Case-control study	Yes	Unknown	Averagely 64.7	8
